# An Efficient Data-Hiding Scheme Based on Multidimensional Mini-SuDoKu

**DOI:** 10.3390/s20092739

**Published:** 2020-05-11

**Authors:** Ji-Hwei Horng, Shuying Xu, Ching-Chun Chang, Chin-Chen Chang

**Affiliations:** 1 Department of Electronic Engineering, National Quemoy University, Kinmen 89250, Taiwan; horng@email.nqu.edu.tw; 2 Department of Information Engineering and Computer Science, Feng Chia University, Taichung 40724, Taiwan; alan3c@gmail.com; 3 Department of Electronic Engineering, Tsinghua University, Beijing 100084, China; c.c.chang.phd@gmail.com; 4 School of Computer Science and Technology, Hangzhou Dianzi University, Hangzhou 310018, China

**Keywords:** data hiding, multidimensional, embedding efficiency, mini-SuDoKu, security

## Abstract

The massive Internet of Things (IoT) connecting various types of intelligent sensors for goods tracking in logistics, environmental monitoring and smart grid management is a crucial future ICT. High-end security and low power consumption are major requirements in scaling up the IoT. In this research, we propose an efficient data-hiding scheme to deal with the security problems and power saving issues of multimedia communication among IoT devises. Data hiding is the practice of hiding secret data into cover images in order to conceal and prevent secret data from being intercepted by malicious attackers. One of the established research streams of data-hiding methods is based on reference matrices (RM). In this study, we propose an efficient data-hiding scheme based on multidimensional mini-SuDoKu RM. The proposed RM possesses high complexity and can effectively improve the security of data hiding. In addition, this study also defines a range locator function which can significantly improve the embedding efficiency of multidimensional RM. Experimental results show that our data-hiding scheme can not only obtain better image quality, but also achieve higher embedding capacity than other related schemes.

## 1. Introduction

We live in the information age in which no such immense amounts of digital information have ever before been consistently transmitted over open communication channels. As a consequence, information security has become a research hotspot. Today, there are two types of strategies for securing information against unauthorized access during transmission. The first type of methods encrypts data by cryptographic algorithms such as RSA [1], DES [2], elliptic-curve signcryption [3] and the blockchain-based solution [4]. However, the use of encryption would easily attract the attention of malicious attackers, causing them to intercept the encrypted data and subsequently use computers of sufficient power to break the encryption [5]. By contrast, the second type of methods hides secret data into cover images by steganographic algorithms and therefore conceals the existence of secret data [6]. After the recipient obtains the stego images, the secret data can be decoded through the corresponding algorithm. Steganographic methods can effectively prevent the interception of the secret data because they conceal the very fact that secret data exists. Indeed, this type of methods has attracted an increasing amount of research attention.

Most data-hiding schemes are performed in the following three domains: frequency domain [7,8], compression domain [9,10,11,12] and spatial domain [13,14,15,16]. Most developers are devoted to devise data-hiding schemes in the spatial domain due to its explicitness and convenience for implementations [17,18,19]. For spatial domain-based data-hiding schemes, reference matrices (RM), as a means of modifying pixels, can achieve low distortion and high embedding capacity. The concept of RM originated from the exploiting modification-direction (EMD) scheme proposed by Zhang and Wang [16] in 2006. Kim et al. proposed an improved version called EMD-2, which modifies the value of up to two pixels in a unit [14]. Compared to the original EMD scheme, this scheme improves the embedding capacity while ensuring the image quality. As another follow-up work, Chang et al. utilized SuDoKu tables as the RM [20]. In this scheme, each pixel pair in the cover image can hide a 9-ary binary secret data, which greatly increases the hiding capacity. Hong et al. [21] proposed a scheme that calculates the distance between pixels by the nearest Euclidean distance, which obtained even better image quality. Turtle shell-based RM is characterized by hexagon shaped shells and is able to hide 3 bits of secret information per pixel [22]. Liu et al. classified the locations on the turtle shell matrix into 16 situations to further improve the embedding capacity with the aid of a location table [23]. Jin et al. combined the data-hiding schemes of the turtle shell and swarm optimization algorithm to improve the visual quality of the image [24]. Our method is inspired by He et al.’s mini-SuDoKu matrix (MSM) [25]. More details of related works will be presented in the following section.

In this study, we propose a 3D RM based on the MSM. Using the 3D-MSM for data hiding can hide more secret data while ensuring image quality. The main contributions of this study are as follows: First, this study proposes a novel 3D reference matrix based on the MSM matrix. Second, it achieves good image quality and embedding capacity. Third, an efficient algorithm is devised to embed secret data. Finally, the proposed algorithm can be generalized to RM of arbitrary N dimensions.

The rest of this study is organized as follows: Section 2 briefly introduces two data-hiding algorithms based on reference matrices. Section 3 introduces the 3D MSM proposed in this study, presents the embedding process, and analyzes the time efficiency of the proposed algorithm. Section 4 compares the proposed scheme with other RM-based data-hiding schemes.

## 2. Related Work

Recent RM-based data-hiding schemes can be characterized into turtle shell-based schemes and SuDoKu-based schemes. In order to pave the way for our idea of multidimensional mini-SuDoKu RM, these two types of matrices are briefly reviewed.

### 2.1. Turtle Shell Matrix Data Hiding

**Figure 1:**
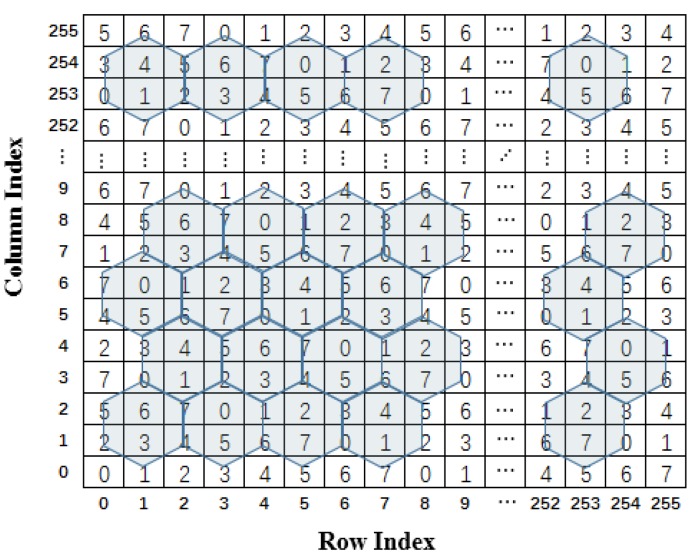
Turtle shell reference matrix.

**Figure 2:**
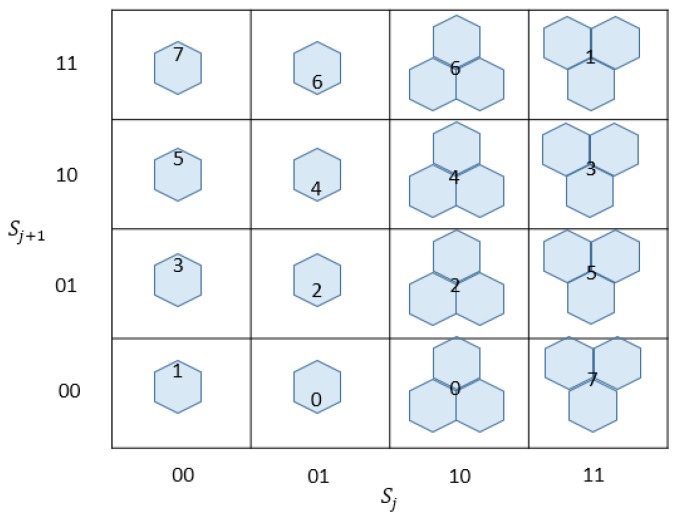
Location table T.

**Figure 3:**
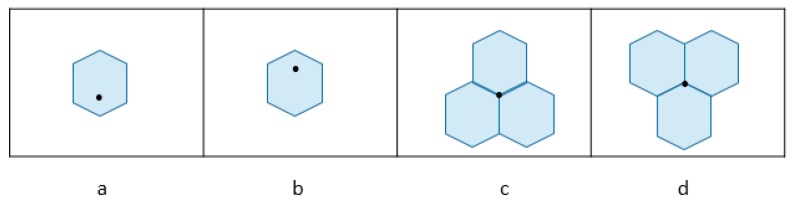
Four locations of elements in M.

In the turtle shell-based scheme proposed by Liu et al. [23], the turtle shell matrix $M=\left[m{\left(i,j\right)}_{i,j\in \left\{0,1,\cdots ,255\right\}}\right]$ is consisted of a number of hexagons, called turtle shells, with a size of 256 × 256, as shown in Figure 1. The RM is filled with 8-ary digits, the incremental value of each row is always 1, while the incremental value of each column change of 2 and 3 in turn. Hence, each turtle shell structure in Figure 1 contains distinct values from 0 to 7. In order to further improve the hiding capacity, a location table is constructed as shown in Figure 2. The location table T contains all 16 possible situations of turtle shell in the RM. The 16 situations in the location table can be grouped into four categories, as shown in Figure 3. According to the characteristics of the RM, the set of values of elements matching location 1 and location 4 is always {1,3,5,7} and matching location 2 and location 3 is always {0,2,4,6}. Each location in the location table T can be represented by $T\left(s_{i},s_{i+1}\right)$, where $s_{i}$ indicates the i-th row, and $s_{i+1}$ indicates the (i+1)-th column, and $s_{i}$ and $s_{i+1}$ belong to {00,01,10,11}.

Next, the process of data hiding is described below. First, the original image is cut into pixel pairs $\left(P_{i},P_{i+1}\right)$, and the binary secret data stream is cut into two sub-streams $s_{j}\;\mathrm{and}\;s_{j+1}$, each of which contains a 2-bit binary number. Second, a pair of cover pixels $\left(P_{i},P_{i+1}\right)$ is applied to locate an element$\;m\left(P_{i},P_{i+1}\right)$ in the RM. After that, employ $\left(s_{j},s_{j+1}\right)$ as coordinates to find the corresponding $T\left(s_{j},s_{j+1}\right)$ in the location table. Then, find the closest element, making $m\left({P_{i}}^{\prime },{P_{i+1}}^{\prime }\right)=T\left(s_{j},s_{j+1}\right)$. Finally, modify the values of the pixel pair to embed it.

By taking relative location of a number in the turtle shell into account, this data-hiding scheme improves the embedding capacity of the original turtle shell scheme from 1.5 to 2. However, the image quality is degraded due to increase of embedding area.

### 2.2. Mini SuDoKu Matrix-Based Data Hiding

The SuDoKu is a matrix which contains nine 3 × 3 sub-matrices with numbers from 1 to 9. In addition, each number is used only once in each raw and each column. Inspired by the conventional SuDoKu, the mini-SuDoKu matrix (MSM) [25] was proposed.

**Figure 4:**
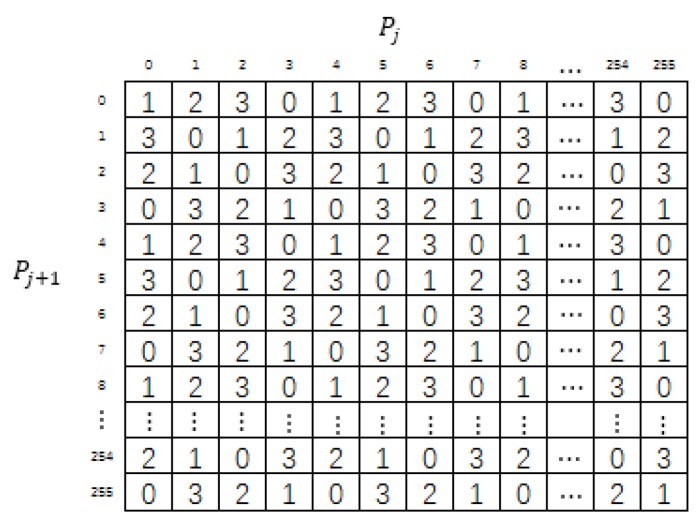
Mini-SuDoKu reference matrix.

As shown in Figure 4, the MSM is a matrix that contains 4096 4 × 4 submatrices, and each submatrix contains 4 basic structures. Each basic structure is filled with numbers from 0 to 3. In addition, the digits from 0 to 3 must occur just once in each row and each column of the submatrix.

The data-hiding scheme using MSM is briefly described as follows: First, the original image is cut into pixel pairs$\;\left(p_{k},p_{k+1}\right)$. The secret data stream is cut into four-bit substreams $sg_{d}=\left\{s_{4d-3},s_{4d-2},s_{4d-1},s_{4d}\right\}$, and then we divide $sg_{d}$ into three groups, i.e., $B_{1}=s_{4d-3}s_{4d-2},B_{2}=s_{4d-1},B_{3}=s_{4d}$. Thus, $B_{1}$ is a quaternary digit from 0 to 3 and $B_{2}$/$B_{3}$ is a bit of 0 or 1. Second, take the pixel pair as coordinates and locate $MSM\left(p_{k},p_{k+1}\right)$ in the MSM. Then, a 4 × 4 candidate block G is determined by (1). Find the element$\;MSM\left({p_{k}}^{\prime },{p_{k+1}}^{\prime }\right)$ within G satisfying $m\left({p_{k}}^{\prime },{p_{k+1}}^{\prime }\right)=B_{1}$,$\;mod\left(p_{k}^{\prime },2\right)=B_{2}$ and$\;mod\left(p_{k+1}^{\prime },2\right)=B_{3}$. Finally, modify the pair $\left(p_{k},p_{k+1}\right)$ to$\;\left({p_{k}}^{\prime },{p_{k+1}}^{\prime }\right)$. We can embed the secret data and get the stego image by repeating these steps.

(1) $$G=\{\begin{array}{c} MSM\left(0:3,0:3\right),\;\mathrm{if}\;p_{k}\leq 1\;\mathrm{and}\;p_{k+1}\leq 1;\\ MSM\left(0:3,p_{k+1}-2:p_{k+1}+1\right),\;\mathrm{if}\;p_{k}\leq 1\;\mathrm{and}\;1<p_{k+1}<255;\\ MSM\left(0:3252:255\right),\;\mathrm{if}\;p_{k}\leq 1\;\mathrm{and}\;p_{k+1}=255;\\ MSM\left(p_{k}-2:p_{k}+1,0:3\right),\;\mathrm{if}\;1<p_{k}<255\;\mathrm{and}\;p_{k+1}\leq 1;\\ MSM\left(p_{k}-2:p_{k}+1,p_{k+1}-2:p_{k+1}+1\right),\;\mathrm{if}\;1<p_{k}<255\;\mathrm{and}\;1<p_{k+1}<255;\\ MSM\left(p_{k}-2:p_{k}+1,p_{k+1},252:255\right),\;\mathrm{if}\;1<p_{k}<255\;\mathrm{and}\;p_{k+1}=255;\\ MSM\left(252:255,0:3\right),\;\mathrm{if}\;p_{k}=255\;\mathrm{and}\;p_{k+1}\leq 1;\\ MSM\left(252:255,p_{k+1}-2:p_{k+1}+1\right),\;\mathrm{if}\;p_{k}=255\;\mathrm{and}\;1<p_{k+1}<255;\\ MSM\left(252:255,252:255\right),\;\mathrm{otherwise} \end{array}$$

**Figure 5:**
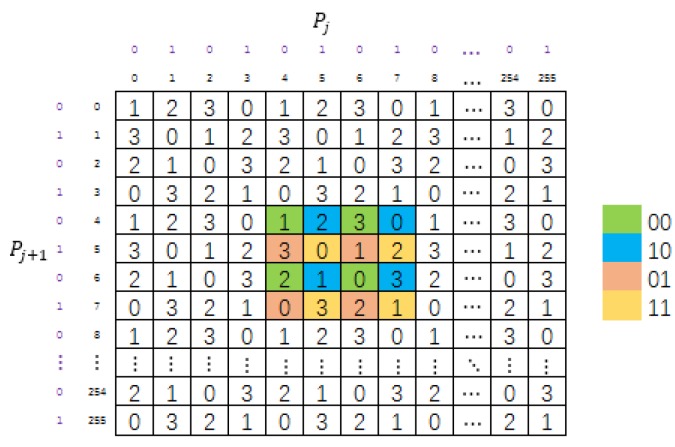
Candidates of embedding for different combinations of $B_{2}$ and $B_{3}$.

The mini-SuDoKu matrix suffers from the problem of low security level. There are too many constraints on the construction rules. Each row, each column and each basic structure of size 2 × 2 must contain distinct values of 0 to 3. To obtain a better PSNR (peak signal to noise ratio) performance, the candidate block for a regular element is defined as $MSM\left(p_{k}-2:p_{k}+1,p_{k+1}-2:p_{k+1}+1\right)$. The two axial ranges of the candidate block do not always coincide with an original 4×4 submatrix. To satisfy the translational invariant requirement for embedding, the whole MSM should repeat the same 4 × 4 submatrix. In addition, the candidates of embedding should satisfy $mod\left(p_{k}^{\prime },2\right)=B_{2}$ and$\;mod\left(p_{k+1}^{\prime },2\right)=B_{3}$. An example grouping of embedding candidates for different combinations of $B_{2}$ and $B_{3}$ is shown in Figure 5. The elements in the same group also should contain all values of 0 to 3. These requirements severely restrict the variety of the MSM and thus threaten the security of data hiding.

## 3. The Proposed Scheme

In this section, we will introduce the proposed cubic mini-SuDoKu matrix and a two-layered data-hiding scheme based on the proposed matrix. Then, the matrix and its corresponding data-embedding and extraction algorithm will be generalized to n-dimensional version. Some mechanisms for improving the time efficiency will also be presented.

### 3.1. Cubic Mini-SuDoKu Matrix (CMSM)

In this study, we propose a two-layered hiding scheme based on a cubic mini-SuDoKu matrix. By leveraging the proposed cubic mini-SuDoKu matrix, the proposed data-hiding scheme can embed secret data with an efficient way and produce stego images of good visual quality.

#### 3.1.1. Construction of the Cubic Mini-SuDoKu Matrix

**Figure 6:**
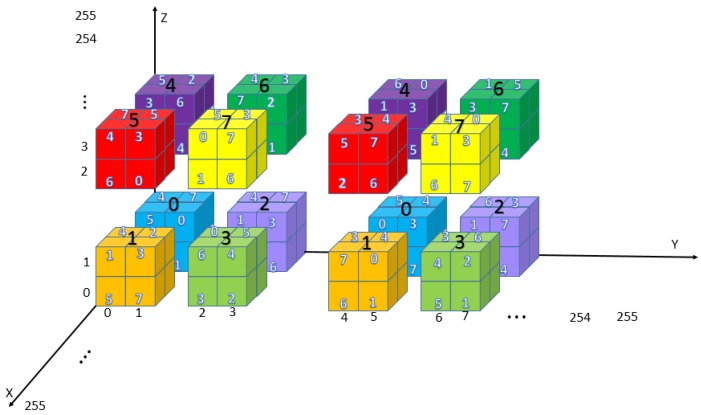
Architecture of cubic matrix.

The cubic mini-SuDoKu matrix is a 256 × 256 × 256 matrix that contains 64 × 64 × 64 sub-cubes of size 4 × 4 × 4. Each sub-cube contains eight basic structures of size 2 × 2 × 2. The basic structures are labeled with bold Arabic numerals as shown in Figure 6. Elements of each basic structure are randomly assigned with distinct values of 0 to 7. The resulting RM is denoted as M(x,y,z), x,y,z=0,1,…,255.

Before embedding, secret data and cover image should be prepared. Binary secret stream is divided into segments of 6 digits each, while the pixels of the cover image are grouped into triplets. A set of three pixels in a triplet $\left(p_{xi},p_{yi},p_{zi}\right)$ is used to embed a secret segment of 6 digits $s_{j}=\left(d_{5}^{j}d_{4}^{j}d_{3}^{j}d_{2}^{j}d_{1}^{j}d_{0}^{j}\right)$. First, the values of the three cover pixels are applied as the coordinates to locate a reference element in the 3D RM. Then, a two-layered embedding scheme is executed. The outer layer is to obtain a matched basic structure by using the three most significant bits (3 MSBs) of secret segment $s_{j}^{M}=\left(d_{5}^{j}d_{4}^{j}d_{3}^{j}\right)$. Subsequently, the inner layer of embedding is to find an element within the obtained basic structure with a value matching the three least significant bits (3 LSBs) $s_{j}^{L}=\left(d_{2}^{j}d_{1}^{j}d_{0}^{j}\right)$. Finally, the pixel values are modified to the indices of the matched element.

To improve the efficiency of outer layer embedding, we define a range locator function G to identify the precise searching range for each direction of axis. By combining ranges of all axes, the basic structure matching the 3 MSBs can be determined.

**Figure 7:**
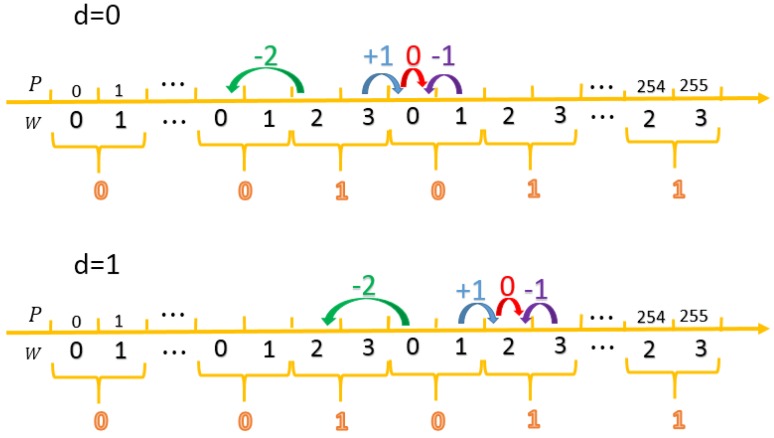
Illustration of incremental value Δ for different situations.

Let p be the pixel value and w=mod(p,4) as shown in Figure 7. We always apply the segment [0:1] of w to embed secret digit d=0, while apply the segment [2:3] of w to embed d=1. To meet this constraint and minimize the modification distortion, we choose the nearest formal segment to embed. For convenience, we define a range front matrix Δ to record the offset values from the current pixel to the range front of embedding. In the case of d=0, the offset is −2 for mod(p,4)=2; +1 for mod(p,4)=3; 0 and −1 for mod(p,4)=0 and 1, respectively. In the case of d=1, the offset is −2 for mod(p,4)=0; +1 for mod(p,4)=1; 0 and −1 for mod(p,4)=2 and 3, respectively. The resulting offset matrix Δ and range locator function G for the entire axis are given in Equations (2) and (3), respectively.

(2) $$\Delta =\left[\begin{array}{cc} \begin{array}{cc} 0 & -1\\ -2 & +1 \end{array} & \begin{array}{cc} -2 & +1\\ 0 & -1 \end{array} \end{array}\right]$$

(3) $$G\left(p,d\right)=\{\begin{array}{l} \;\;\;\;\;\;\;\;\;\;\;\;\;\left[0:1\right],\;\mathrm{if}\;p=0\;\mathrm{and}\;d=0;\\ \;\;\;\;\;\;\;\;\;\;\;\;\;\left[2:3\right],\;\mathrm{if}\;p=0\;\mathrm{and}\;d=1;\\ \;\;\;\;\;\;\;\;\;\;\;\;\left[252:253\right],\;\mathrm{if}\;p=255\;\mathrm{and}\;d=0;\\ \;\;\;\;\;\;\;\;\;\;\;\;\left[254:255\right],\;\mathrm{if}\;p=255\;\mathrm{and}\;d=1;\\ \left[p^{\prime }:p^{\prime }+1\right]\;\mathrm{with}\;p^{\prime }=p+\Delta \left(d,mod\left(p,4\right)\right),\;\mathrm{if}\;1\leq p\leq 254. \end{array}$$

**Figure 8:**
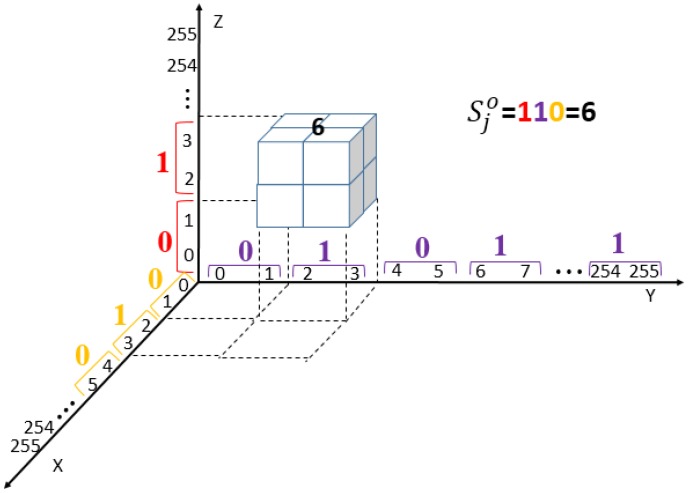
Locating the basic structure for embedding by combining ranges of three axes.

Figure 8 illustrates an example of combining ranges of three axes. Assuming the cover triplet is $\left(p_{xi},p_{yi},p_{zi}\right)=\left(2,3,2\right)$ and the 3 MSBs of the secret segment to be embedded is $s_{j}^{M}=\left(d_{5}^{j}d_{4}^{j}d_{3}^{j}\right)={\left(110\right)}_{2}$. By applying the range locater, the embedding range of each axis can be determined independently as $G\left(p_{xi},d_{3}^{j}\right)=G\left(2,0\right)=\left[2+\Delta \left(0,2\right):2+\Delta \left(0,2\right)+1\right]=\left[0:1\right]$, $G\left(p_{yi},d_{4}^{j}\right)=G\left(3,1\right)=\left[3+\Delta \left(1,3\right):3+\Delta \left(1,3\right)+1\right]=\left[2:3\right]$ and $G\left(p_{zi},d_{5}^{j}\right)=G\left(2,1\right)=\left[2+\Delta \left(1,2\right):2+\Delta \left(1,2\right)+1\right]=\left[2:3\right]$. As shown in the figure, the basic structure obtained by combining the located ranges is M(0:1,2:3,2:3). Comparing with Figure 6, the basic structure obtained by applying $s_{j}^{M}={\left(110\right)}_{2}$ to range locater coincides with the structure labeled 6$={\left(110\right)}_{2}$. This result demonstrates that the range locater can be treated as an efficient tool for the outer layer of embedding scheme.

#### 3.1.2. Secret Data Embedding

As mentioned in the previous subsection, the proposed data-embedding scheme is composed of two hiding layers. The outer layer uses the 3 MSBs to locate the nearest formal basic structure for embedding. The inner layer seeks to find the element with a value matching the 3 LSBs and embeds the 6 digits in total by modifying the pixel values. The details of secret data embedding are as follows:

  The secret data-embedding algorithm based on the cubic mini-SuDoKu matrix (CMSM)

Input: cover image P, secret stream S, secret key *K*

Output: stego image $P^{\prime }$ Step 1: Construct the CMSM M using the secret key *K* (details are given in Appendix A)

(a)  Apply the secret key *K* to initialize the random number generator;

(b)  Allocate an empty array of size 256×256×256 and divide it into blocks of size 2×2×2;

(c)  Fill in each block with random ordered 0 to 7, consecutively;

Step 2: Group the cover pixels into triplets $P=\left\{\left(p_{xi},p_{yi},p_{zi}\right)|i=1,\;2,\;\ldots ,\;\left(H\times W\right)/3\right\}$;

Step 3: Segment secret digits $S=\left\{s_{j}=\left(d_{5}^{j}d_{4}^{j}d_{3}^{j}d_{2}^{j}d_{1}^{j}d_{0}^{j}\right)|j=1,\;2,\;\ldots ,L/6\right\}$;

Step 4: Locate $M\left(G\left(p_{xi},d_{3}^{j}\right),G\left(p_{yi},d_{4}^{j}\right),G\left(p_{zi},d_{5}^{j}\right)\right)$ by applying Equations (2) and (3);

Step 5: Search the matching element in the located basic structure;

$M\left(p_{xi}^{\prime },p_{yi}^{\prime },p_{zi}^{\prime }\right)=\left(2^{2}\times d_{2}^{j}+2^{1}\times d_{1}^{j}+2^{0}\times d_{0}^{j}\right)$;

Step 6: Record $\left(p_{xi}^{\prime },p_{yi}^{\prime },p_{zi}^{\prime }\right)$ to stego image $P^{\prime }$;

Step 7: Repeat Steps 4–6, until all secret digits are embedded.

In the embedding algorithm, we use a secret key *K* to initialize the random number generator. Each basic structure is stored with a random permutation of 0 to 7. The number of different CMSM is ${\left(8!\right)}^{128\times 128\times 128}$. To reduce the computational load of the matrix, we can produce a randomly generated matrix of size, for example, 16 × 16 × 16 and repeat it to obtain a 256 × 256 × 256 CMSM. The number of different permutations is ${\left(8!\right)}^{16\times 16\times 16}$, which is still much secure than the 2D mini-SuDoKu version [23]. By sharing the initialization key for the random number generator, the receiver can reconstruct the CMSM using the same rule.

**Figure 9:**
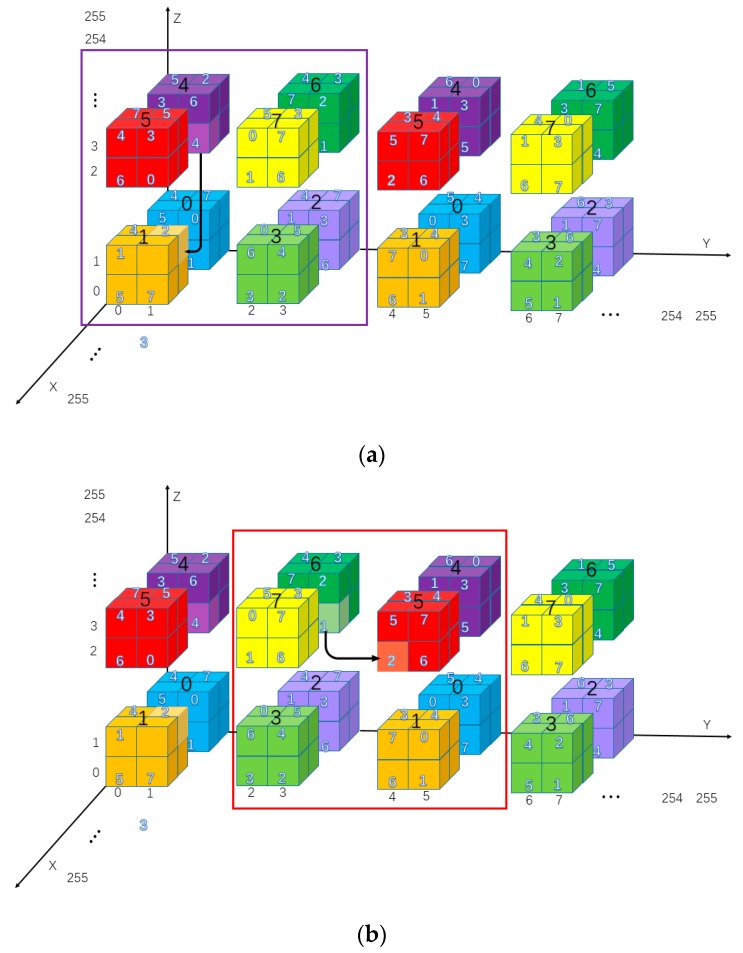

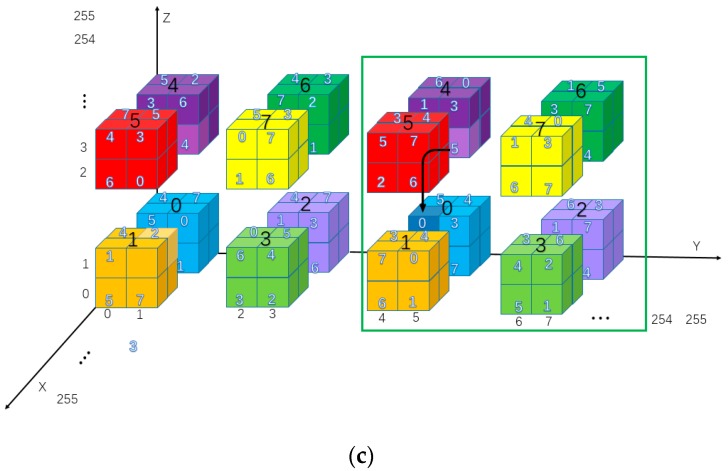
Examples of data hiding with cubic mini-SuDoKu matrix (CMSM). (**a**) Hide ${\left(001010\right)}_{2}$ into cover triplet (1,1,2); (**b**) Hide ${\left(101010\right)}_{2}$ into cover triplet (1,3,2); (**c**) Hide ${\left(000000\right)}_{2}$ into cover triplet (1,5,2).

In the following, three examples are used to further explain the secret data-embedding process as shown in Figure 9. Assume the three triplets are {(1,1,2),(1,3,2),(1,5,2)}, which will be used to hide the secret segments {${\left(001010\right)}_{2},{\left(101010\right)}_{2},{\left(000000\right)}_{2}$}.

For the triplet (1,1,2) as shown by the purple submatrix in Figure 9a, the secret data $s_{j}=\left(d_{5}^{j}d_{4}^{j}d_{3}^{j}d_{2}^{j}d_{1}^{j}d_{0}^{j}\right)={\left(001010\right)}_{2}$ is to be hidden. First, use triplet (1,1,2) and 3 MSBs $s_{j}^{M}={\left(001\right)}_{2}$ to locate the basic structure $G\left(p_{xi},d_{3}^{j}\right)=G\left(1,1\right)=\left[1+\Delta \left(1,1\right):1+\Delta \left(1,1\right)+1\right]=\left[2:3\right]$, $G\left(p_{yi},d_{4}^{j}\right)=G\left(1,0\right)=\left[1+\Delta \left(0,1\right):1+\Delta \left(0,1\right)+1\right]=\left[0:1\right]$, $G\left(p_{zi},d_{5}^{j}\right)=G\left(2,0\right)=\left[2+\Delta \left(0,2\right):2+\Delta \left(0,2\right)+1\right]=\left[0:1\right]$, and $M\left(G\left(p_{xi},d_{3}^{j}\right),G\left(p_{yi},d_{4}^{j}\right),G\left(p_{zi},d_{5}^{j}\right)\right)=M\left(2:3,0:1,0:1\right)$. Then, $M\left(2,1,1\right)=2^{2}\times 0+2^{1}\times 1+2^{0}\times 0=2$ can be found in the located basic structure and record (2,1,1) to stego image $P^{\prime }$.

For the second example of (1,3,2) as shown by the red submatrix in Figure 9b, the secret data $s_{j}=\left(d_{5}^{j}d_{4}^{j}d_{3}^{j}d_{2}^{j}d_{1}^{j}d_{0}^{j}\right)={\left(101010\right)}_{2}$ is to be hidden. First, use triplet (1,3,2) and 3 MSBs $s_{j}^{M}={\left(101\right)}_{2}$ to locate the basic structure $G\left(p_{xi},d_{3}^{j}\right)=G\left(1,1\right)=\left[1+\Delta \left(1,1\right):1+\Delta \left(1,1\right)+1\right]=\left[2:3\right]$, $G\left(p_{yi},d_{4}^{j}\right)=G\left(3,0\right)=\left[3+\Delta \left(0,3\right):3+\Delta \left(0,3\right)+1\right]=\left[4:5\right]$, $G\left(p_{zi},d_{5}^{j}\right)=G\left(2,1\right)=\left[2+\Delta \left(1,2\right):2+\Delta \left(1,2\right)+1\right]=\left[2:3\right]$, and $M\left(G\left(p_{xi},d_{3}^{j}\right),G\left(p_{yi},d_{4}^{j}\right),G\left(p_{zi},d_{5}^{j}\right)\right)=M\left(2:3,4:5,2:3\right)$. Then, $M\left(3,4,2\right)=2^{2}\times 0+2^{1}\times 1+2^{0}\times 0=2$ can be found in the located basic structure and record (3,4,2) to stego image $P^{\prime }$.

For the third triplet (1,5,2) as shown by the green submatrix in Figure 9c, the secret data $s_{j}=\left(d_{5}^{j}d_{4}^{j}d_{3}^{j}d_{2}^{j}d_{1}^{j}d_{0}^{j}\right)={\left(000000\right)}_{2}$ is to be hidden. First, use triplet (1,5,2) and 3 MSBs $s_{j}^{M}={\left(000\right)}_{2}$ to locate the basic structure$\;G\left(p_{xi},d_{3}^{j}\right)=G\left(1,0\right)=\left[1+\Delta \left(0,1\right):1+\Delta \left(0,1\right)+1\right]=\left[0:1\right]$, $G\left(p_{yi},d_{4}^{j}\right)=G\left(5,0\right)=\left[5+\Delta \left(0,5\right):5+\Delta \left(0,5\right)+1\right]=\left[4:5\right]$, $G\left(p_{zi},d_{5}^{j}\right)=G\left(2,0\right)=\left[2+\Delta \left(0,2\right):2+\Delta \left(0,2\right)+1\right]=\left[0:1\right]$, and $M\left(G\left(p_{xi},d_{3}^{j}\right),G\left(p_{yi},d_{4}^{j}\right),G\left(p_{zi},d_{5}^{j}\right)\right)=M\left(0:1,4:5,0:1\right)$. Then, $M\left(1,4,1\right)=2^{2}\times 0+2^{1}\times 0+2^{0}\times 0=0$ can be found in the located basic structure and record (1,4,1) to stego image $P^{\prime }$.

#### 3.1.3. Secret Data Extraction

After receiving the stego image $P^{\prime }$, the recipient first groups the stego pixels into triplets. Then, the secret segments can be obtained by mapping the triplets into the CMSM. For a located element, its corresponding secret segment includes the 3 MSBs determined by the label of basic structure it belongs to and the 3 LSBs determined by its value. The details of the extraction process are provided as follows:

The secret data extraction algorithm based on the cubic mini-SuDoKu matrix (CMSM)

Input: stego image $P^{\prime }$, secret key *K*

Output: secret stream S Step 1: Construct the CMSM M using the secret key *K* (details are given in Appendix A)

(a)  Apply the secret key *K* to initialize the random number generator.

(b) Allocate an empty array of size 256×256×256 and divide it into blocks of size 2×2×2.

(c) Fill in each block with random ordered 0 to 7, consecutively.

Step 2: Group the stego pixels into triplets $P=\left\{\left(p_{xi}^{\prime },p_{yi}^{\prime },p_{zi}^{\prime }\right)|i=1,\;2,\;\ldots ,\;\left(H\times W\right)/3\right\}$.

Step 3: Extract the 3 LSBs by $s_{j}^{L}={\left[M\left(p_{xi}^{\prime },p_{yi}^{\prime },p_{zi}^{\prime }\right)\right]}_{2}$.

Step 4: Extract the 3 MSBs by

  $s_{j}^{M}=\left(d_{5}^{j},d_{4}^{j},d_{3}^{j}\right)=\left(mod(p_{zi}^{\prime },4)/2,mod(p_{yi}^{\prime },4)/2,mod(p_{xi}^{\prime },4)/2\right)$.

Step 5: Concatenate $s_{j}=s_{j}^{M}s_{j}^{L}$.

Step 6: Repeat Steps 3–5, until all secret digits are extracted.

### 3.2. N-Dimensional MSM (NMSM)

In this section, we introduce the construction of NMSM and the secret data-embedding and extraction algorithm, based on the NMSM. In addition, a fast algorithm for the inner embedding layer is proposed to improve the time efficiency.

#### 3.2.1. The Data-Embedding and Extraction Algorithm

To boost the efficiency of data embedding and extraction, the proposed secret data-embedding scheme can be generalized to an n-dimensional version. In the NMSM, a basic structure consists of $2^{n}$ elements and $2^{n}$ basic structures constitute a submatrix. An n-tuple pixel group can uniquely map to an element in the NMSM. Therefore, by applying the same embedding rule, we can hide n MSBs with the label of basic structure and n LSBs with the element value. The pseudo codes for the NMSM-based embedding and extraction schemes are given as follows:

The secret data-embedding algorithm based on the n-dimensional mini-SuDoKu matrix (NMSM)

Input: cover image P, secret stream S, secret key *K*

Output: stego image $P^{\prime }$ Step 1: Construct the NMSM M using the secret key *K* (details are given in Appendix B);

(a) Make an n-dimensional array of size $16^{n}$, which consists of $8^{n}$ submatrix;

(b) Repeat the array to obtain NMSM;

Step 2: Group the cover pixels into $P=\left\{\left(p_{X\left(0\right)i},p_{X\left(1\right)i},\ldots ,p_{X\left(n-1\right)i}\right)|i=1,\;2,\;\ldots ,\;\left(H\times W\right)/n\right\}$;

Step 3: Segment secret digits $S=\left\{s_{j}=\left(d_{2n-1}^{j}d_{2n-2}^{j}\ldots d_{0}^{j}\right)|j=1,\;2,\;\ldots ,L/2n\right\}$;

Step 4: Locate the basic structure$\;M\left(G\left(p_{X\left(0\right)i},d_{n}^{j}\right),G\left(p_{X\left(1\right)i},d_{n+1}^{j}\right),\ldots ,G\left(p_{X\left(n-1\right)i},d_{2n-1}^{j}\right)\right)$

  by applying Equations (2) and (3);

Step 5: Search the matching element in the located basic structure

  $M\left(p_{X\left(0\right)i}^{\prime },p_{X\left(1\right)i}^{\prime },\ldots ,p_{X\left(n-1\right)i}^{\prime }\right)=\left(2^{n-1}\times d_{n-1}^{j}+2^{n-2}\times d_{n-2}^{j}+\ldots +2^{0}\times d_{0}^{j}\right)$;

Step 6: Record $\left(p_{X\left(0\right)i}^{\prime },p_{X\left(1\right)i}^{\prime },\ldots ,p_{X\left(n-1\right)i}^{\prime }\right)$ to stego image $P^{\prime }$;

Step 7: Repeat Steps 4–6, until all secret digits are embedded.

The secret data extraction algorithm based on the n-dimensional mini-SuDoKu matrix (NMSM)

Input: stego image $P^{\prime }$, secret key *K*

Output: secret stream S

Step 1: Construct the NMSM by the same process as Step 1 of embedding;

Step 2: Group the stego pixels into $P^{\prime }=\left\{\left(p_{X\left(0\right)i}^{\prime },p_{X\left(1\right)i}^{\prime },\ldots ,p_{X\left(n-1\right)i}^{\prime }\right)|i=1,\;2,\;\ldots ,\;\left(H\times W\right)/n\right\}$;

Step 3: Extract the n LSBs by $s_{j}^{L}={\left[M\left(p_{X\left(0\right)i}^{\prime },p_{X\left(1\right)i}^{\prime },\ldots ,p_{X\left(n-1\right)i}^{\prime }\right)\right]}_{2}$;

Step 4: Extract the n MSBs by

    $s_{j}^{M}=\left(mod(p_{X\left(n-1\right)i}^{\prime },4)/2,mod(p_{X\left(n-2\right)i}^{\prime },4)/2,\ldots ,mod(p_{X\left(0\right)i}^{\prime },4)/2\right)$;

Step 5: Concatenate $s_{j}=s_{j}^{M}s_{j}^{L}$;

Step 6: Repeat Steps 3–5, until all secret digits are extracted.

#### 3.2.2. Fast Algorithm for the Inner Layer of Embedding

As the MSM generalized to n-dimensions, the basic structure for embedding can be efficiently determined by the range locator. However, the searching process in the Step 5 of embedding algorithm becomes burdensome. To improve time efficiency of the inner embedding layer, we devise a fast algorithm to overcome this burden. Its key idea is to leverage the matrix operation supported by the MATLAB language. The pseudo code of the fast algorithm is given as follows: More precise pseudo code expressed in MATLAB instructions is given in Appendix C.

Fast Algorithm for the Inner Embedding Layer (details are given in Appendix C)

Input: n LSBs of secret segment $s_{j}^{L}$, basic structure for embedding

$$A=M\left(G\left(p_{X\left(0\right)i},d_{n}^{j}\right),G\left(p_{X\left(1\right)i},d_{n+1}^{j}\right),\ldots ,G\left(p_{X\left(n-1\right)i},d_{2n-1}^{j}\right)\right)$$

Output: stego pixel values $\left(p_{X\left(0\right)i}^{\prime },p_{X\left(1\right)i}^{\prime },\ldots ,p_{X\left(n-1\right)i}^{\prime }\right)$

(a)  Construct an n-dimensional basic structure B with all elements valued with $s_{j}^{L}$;

(b)  Using matrix operation to find the only matched element in both A and B;

(c)  Project the element to all axes and obtain the coordinates for embedding.

## 4. Experimental Results

**Figure 10:**
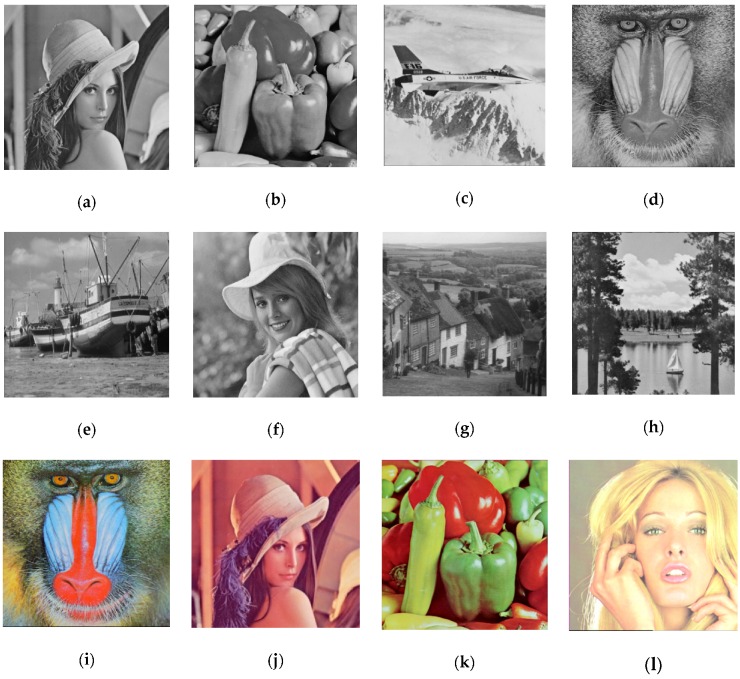
Twelve cover images with size 512 × 512: (**a**) Lena; (**b**) peppers; (**c**) airplane; (**d**) baboon; (**e**) boat; (**f**) Elaine; (**g**) Gledhill; (**h**) sailboat; (**i**) baboon (RGB); (**j**) Lena (RGB); (**k**) peppers (RGB); and (**l**) Tiffany (RGB).

**Figure 11:**
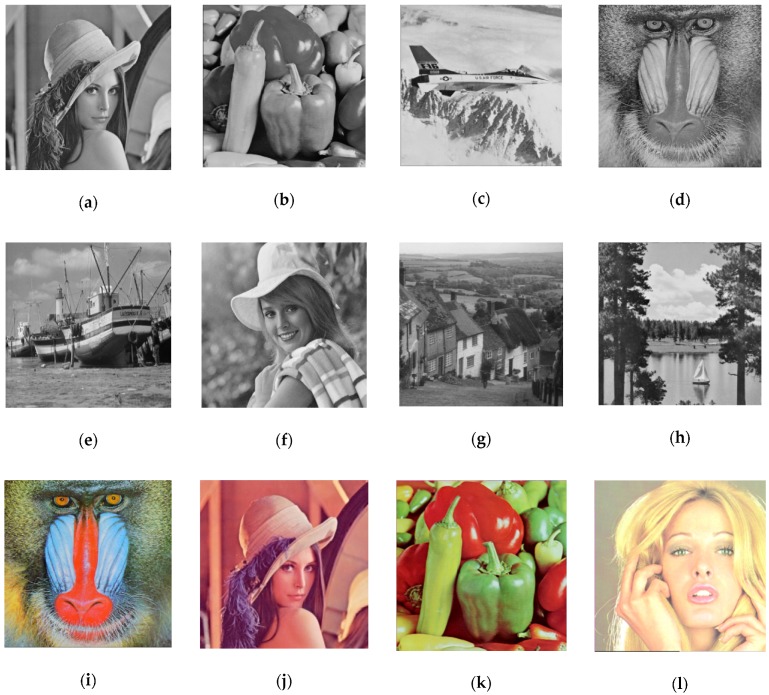
Twelve stego images with size 512 × 512: (**a**) Lena; (**b**) peppers; (**c**) airplane; (**d**) baboon; (**e**) boat; (**f**) Elaine; (**g**) Gledhill; (**h**) sailboat; (**i**) baboon (RGB); (**j**) Lena (RGB); (**k**) peppers (RGB); and (**l**) Tiffany (RGB).

In the following, the experimental results of the proposed scheme will be presented and compared with the related works. As shown in Figure 10, this study uses eight standard 512 × 512 grayscale images and four standard 512 × 512 true color images as the cover image P for secret data embedding. All experiments are implemented with MATLAB R2017b. Figure 11 shows the twelve stego images obtained by applying the two-layered hiding scheme based on the CMSM.

The stego images look like the cover images and cannot be distinguished by human eyes. The peak signal-to-noise ratio (PSNR) is used to measure the quality of a stego image. Its calculation is given by Equation (4). In the equation, P and $P^{\prime }$ represent the cover image and the stego image, respectively, H and W represent their height and width and (m,n) represents the coordinate of the pixel.

(4) $$PSNR=10\times \log_{10}\frac{255^{2}\times H\times W}{\sum_{m=1}^{H}\sum_{n=1}^{W}{\left[P\left(m,n\right)-P^{\prime }\left(m,n\right)\right]}^{2}}.$$

In addition, we also use structural similarity index (SSIM) to measure the similarity between a cover image and its corresponding stego image. Let P and $P^{\prime }$ represent the cover image and the stego image, respectively, the SSIM of the two images can be obtained according to Equation (5), where $\mu_{P}$ is the average of P, $\mu_{P^{\prime }}$ is the average of $P^{\prime }$, $\sigma_{P}^{2}$ is the variance of P, $\sigma_{P^{\prime }}^{2}$ is the variance of $P^{\prime }$ and $\sigma_{PP^{\prime }}$ is the covariance of P and $P^{\prime }$. In addition, $c_{1}$ and $c_{2}$ are constants used to maintain stability and can be obtained by Equations (6) and (7), respectively, where *L* is the dynamic range of pixel values,$\;k_{1}=0.01$, $k_{2}=0.03$.

(5) $$SSIM\left(P,P^{\prime }\right)=\frac{\left(2\mu_{P}\mu_{P^{\prime }}+c_{1}\right)\left(2\sigma_{PP^{\prime }}+c_{2}\right)}{\left(\mu_{P}^{2}+\mu_{P^{\prime }}^{2}+c_{1}\right)\left(\sigma_{P}^{2}+\sigma_{P^{\prime }}^{2}+c_{2}\right)}.$$

(6) $$c_{1}={\left(k_{1}L\right)}^{2}.$$

(7) $$c_{2}={\left(k_{2}L\right)}^{2}.$$

Embedding capacity (EC) is another important issue in the image steganography. It is used to measure the maximum amount of secret data that can be embedded in an image by a data-hiding scheme. Since EC is dependent on the image size, we further define the embedding rate (ER) to express the average number of secret bits that each pixel can embed. ER is defined as (8), where ||S|| represents the total amount of secret data embedded in the entire stego image.

(8) $$ER=\frac{\left|\left|S\right|\right|}{M\times N}.$$

Table 1 shows the experimental results of the grayscale images. In our hiding scheme, three cover pixels are applied to embed a secret segment of six digits. Therefore, the ER measure is 6 bits/3 pixels = 2 bits/pixel, the embedding capacity for an applied grayscale cover image is therefore 512 × 512 × 2 = 524,288 bits as shown in the Under full embedding, the proposed scheme achieves a high image quality of PSNR = 46.37 dB and SSIM = 0.9923 in average. The quality of stego image is irrelevant to features of the cover image. For the true color images, each pixel consists of three channels, including red, green and blue. Each of the three channels is represented by one byte. By mapping the three bytes (r, g,b) of a pixel into the CMSM, we can hide a secret segment of six bits by applying the proposed embedding algorithm. Therefore, the embedding capacity of an applied true color image is 512 × 512 × 6 = 1,572,864 bits. The PSNR and SSIM of the true color stego images are very close to the experimental values of grayscale images as shown in Table 2.

### 4.1. EC and PSNR Comparison

Table 3 compares the PSNR of different reference matrices under the same EC. As shown in Table 3, Xie et al.’s scheme has the lowest average PSNR at 41.87 dB. The average PSNR of the proposed scheme is 46.37 dB, which is nearly 4 dB higher than the average PSNR of the Xie et al.’s scheme. Moreover, compared with the other two schemes, our scheme also achieves the highest PSNR with the same EC. It can be seen that the proposed scheme outperforms the related works.

In order to further understand the performance of our scheme, it is compared with three other schemes [19,22,23] based on the SuDoKu reference matrix. As shown in Table 4, the EC of the Chang et al.’s scheme is 393,216 bits, and its average PSNR is 44.83 dB. Regardless of the EC or image quality, the proposed scheme outperforms the Chang et al.’s scheme. Comparing with the other two schemes, although their image quality are better than the proposed scheme, their embedding capacity are far lower than our scheme. Therefore, it can be concluded that the overall performance of the proposed scheme is better than the SuDoKu-based data-hiding schemes.

In addition, we also compare the proposed CMSM-based scheme with the 3D SuDoKu-based scheme. As shown in Table 5, the average PSNR of the scheme proposed by Xia et al. is 41.31 dB, while the average PSNR of our scheme is 5 dB higher than their scheme. Based on the same frame structure of using 3D reference matrix, our scheme has a relatively small modification of pixel values under the same EC.

Our scheme is inspired by the He et al.’s scheme. Except for expanding the 2D mini-SuDoKu to a 3D CMSM, our scheme effectively improved the complexity of reference matrix and efficiently reduced the computation time. As shown in Table 6, although our scheme has no advantage over the He et al.’s scheme in terms of EC and PSNR. However, the improvement in time consumption (TC) is obvious. As discussed in Section 2.2, to achieve a translation invariant property for minimizing the distortion of pixel value modification, the mini-SuDoKu has to repeat a same basic 4 × 4 submatrix. This severely damages the complexity of a reference matrix. In our scheme, in spite of CMSM or NMSM, each basic structure is a completely random permutation of $2^{n}$ distinct numbers. The possible combinations of a CMSM is an enormous figure. In the construction of NMSM, referring to Section 3.2.1, we even truncate the size of a randomly generated array and make the whole NMSM by repetition to reduce computational load. The key difference with the He et al.’s scheme is the design of range locator. It releases the constraint on the diversity of reference matrix. An additional benefit of the range locator is that the basic structure for embedding can be efficiently located without applying a time-consuming searching process. The cooperation of range locater in the outer embedding and the matrix operation in the inner embedding frees the hiding scheme from intensive loops of searching. According to the experimental data in Table 6, the required embedding time is less than half of the compared scheme.

### 4.2. Time Efficiency Comparison

To investigate the time efficiency of the proposed algorithm, we try to compare the time consumption of the proposed algorithm with the traditional approach. Although we present the three-dimensional (3D) CMSM and the n-dimensional (n−D) NMSM, our approach can also be de-generalized back to two-dimensional (2D) and one-dimensional (1D) version.

This experiment uses 8 typical grayscale images for testing and compares the proposed algorithm with a traditional one. A computer with a Dual i7-920 CPU and 8 GB memory is adopted for the experiment. The tic and toc commands in MATLAB are used to record the time cost in seconds. As shown in Table 7, due to the large number of loops used in the search step of the traditional algorithm, it takes significantly longer time to embed secret data. As the dimension rises, the time consumption increases rapidly. On the other hand, our algorithm has consistent performance as the dimension rises. Note that, as the dimension rises, the complexity of the reference matrix increases and thus the security level raises.

## 5. Conclusions

This study introduces an efficient multidimensional secret data-embedding scheme based on the mini SuDoKu matrix. In the proposed scheme, a CMSM RM with high complexity is first constructed to guarantee the security, and then a range locator function and the matrix operation are adopted to enhance the embedding efficiency. The reference matrix is further expanded to multidimension in order to obtain even higher embedding capacity and, meanwhile, still preserve good security and efficiency. The proposed scheme is compared with state-of-the-art RM-based data-hiding schemes and the experimental results show that the proposed scheme achieved higher than 46 dB in terms of the image quality and two bits per pixel in terms of the embedding capacity. In addition, the time consumption of the proposed algorithm is less than half of the traditional approach and keeps consistency as the dimension and security level raises. It is shown that the proposed scheme is advantageous in both embedding efficiency and security compared to the original mini-SuDoKu matrix.

We also provide a set of true color test images to demonstrate that the proposed scheme performs equal well to multi-channel images. By leveraging CMSM, each pixel of three-color channels—i.e., R, G, and B—can exactly match with the requirement of embedding a secret segment of data. The flexibility in dimension of RM meets the diverse data structure of cover media in the future word of massive IoT.

## Figures and Tables

**Table 1:** Experimental results for grayscale images.

Image	EC (bits)	PSNR (dB)	SSIM
Lena	524,288	46.38	0.9918
Peppers	524,288	46.37	0.9906
Airplane	524,288	46.37	0.9883
Baboon	524,288	46.36	0.9958
Boat	524,288	46.37	0.9938
Elaine	524,288	46.38	0.9919
Gledhill	524,288	46.36	0.9936
Sailboat	524,288	46.37	0.9929
Average	524.288	46.37	0.9923

**Table 2:** Experimental results for color images.

Image	EC (bits)	PSNR (dB)	SSIM
Baboon (RGB)	1,572,864	46.36	0.9930
Lena (RGB)	1,572,864	46.38	0.9926
Peppers (RGB)	1,572,864	46.37	0.9908
Elaine (RGB)	1,572,864	46.37	0.9916
Average (RGB)	1,572,864	46.37	0.9920

**Table 3:** Comparison with related works.

Image	Jin et al. [24]		Liu et al. [23]		Xie et al. [17]		Proposed	
	EC	PSNR	EC	PSNR	EC	PSNR	EC	PSNR
Lena	524,288	45.57	524,288	45.55	524,288	41.87	524,288	46.38
Peppers	524,288	45.56	524,288	45.54	524,288	41.86	524,288	46.37
Airplane	524,288	45.56	524,288	45.58	524,288	41.87	524,288	46.37
Baboon	524,288	45.57	524,288	45.55	524,288	41.86	524,288	46.36
Boat	524,288	45.58	524,288	45.54	524,288	41.87	524,288	46.37
Elaine	524,288	45.56	524,288	45.49	524,288	41.87	524,288	46.38
Gledhill	524,288	45.49	524,288	45.49	524,288	41.87	524,288	46.36
Sailboat	524,288	45.58	524,288	45.55	524,288	41.86	524,288	46.37
Average	524,288	45.56	524,288	45.54	524,288	41.87	524,288	46.37

**Table 4:** Comparison among SuDoKu-based data-hiding schemes.

Image	Chang et al. [20]		Hong et al. [21]		Lin et al. [19]		Proposed	
	EC	PSNR	EC	PSNR	EC	PSNR	EC	PSNR
Lena	393,216	44.96	393,216	48.68	393,216	49.90	524,288	46.38
Peppers	393,216	44.67	393,216	48.67	393,216	49.91	524,288	46.37
Airplane	393,216	44.99	393,216	48.68	393,216	49.92	524,288	46.37
Baboon	393,216	44.68	393,216	48.66	393,216	.49.89	524,288	46.36
Boat	393,216	44.90	393,216	48.67	393,216	49.91	524,288	46.37
Elaine	393,216	44.92	393,216	48.68	393,216	49.91	524,288	46.38
Gledhill	393,216	44.85	393,216	48.67	393,216	49.90	524,288	46.36
Sailboat	393,216	44.67	393,216	48.67	393,216	49.90	524,288	46.37
Average	393,216	44.83	393,216	48.67	393,216	49.90	524,288	46.37

**Table 5:** Comparison with the 3D SuDoKu matrix-based scheme.

Image	Xia et al. [18]		Proposed	
	EC	PSNR	EC	PSNR
Lena	524,288	41.31	524,288	46.38
Peppers	524,288	41.30	524,288	46.37
Airplane	524,288	41.28	524,288	46.37
Baboon	524,288	41.25	524,288	46.36
Boat	524,288	41.23	524,288	46.37
Elaine	524,288	41.26	524,288	46.38
Gledhill	524,288	41.29	524,288	46.36
Sailboat	524,288	41.27	524,288	46.37
Average	524,288	41.27	524,288	46.37

**Table 6:** Comparison with the mini-SuDoKu matrix-based scheme.

Image	He et al. [25]			Proposed		
	EC	PSNR	TC	EC	PSNR	TC
Lena	524,288	46.37	2.71 s	524,288	46.38	1.07 s
Peppers	524,288	46.37	2.72 s	524,288	46.37	1.12 s
Airplane	524,288	46.37	2.70 s	524,288	46.37	1.10 s
Baboon	524,288	46.36	2.73 s	524,288	46.36	1.09 s
Boat	524,288	46.36	2.71 s	524,288	46.37	1.11 s
Elaine	524,288	46.38	2.71 s	524,288	46.38	1.12 s
Gledhill	524,288	46.37	2.72 s	524,288	46.36	1.09 s
Sailboat	524,288	46.35	2.71 s	524,288	46.37	1.12 s
Average	524,288	46.37	2.71 s	524,288	46.37	1.10 s

**Table 7:** Comparison of time efficiency between the proposed scheme and traditional algorithm.

Image	Trad-2D	Trad-3D	Proposed-2D	Proposed-3D	Proposed-4D
Lena	2.80 s	4.48 s	1.09 s	1.07 s	1.11 s
Peppers	2.81 s	4.51 s	1.11 s	1.12 s	1.13 s
Airplane	2.83 s	4.52 s	1.10 s	1.10 s	1.12 s
Baboon	2.82 s	4.49 s	1.11 s	1.09 s	1.11 s
Boat	2.75 s	4.47 s	1.10 s	1.11 s	1.12 s
Elaine	2.94 s	4.54 s	1.13 s	1.12 s	1.14 s
Gledhill	2.86 s	4.54 s	1.08 s	1.09 s	1.11 s
Sailboat	2.83 s	4.49 s	1.11 s	1.13 s	1.13 s

## Appendix A

  The pseudo code for constructing the CMSM is provided as follows:

(a) Apply the secret key to initialize the random number generator.

  rng(*K*);

(b) Allocate an empty array of size 256×256×256 and divide it into blocks of size 2×2×2.

  M=zero(256,256,256);

(c) Fill in each block with random ordered 0 to 7, consecutively;

  for x=0:2:255,

  for y=0:2:255,

  for z=0:2:255,

                        V=randperm(8)−1;

    A=[V(0) V(1);V(2) V(3)]; B=[V(4) V(5);V(6) V(7)];

                     M(x:x+1,y:y+1,z:z+1)=A;

  end

  end

  end

  The MATLAB functions rng() and randperm(8) are used to initialize random number generator and produce random permutations of 1 to 8, respectively. Although we apply the coding format and the functions of MATLAB programming language, the index of an array in the pseudo code follows the convention of zero leading value.

## Appendix B

  The pseudo code for constructing the NMSM is provided as follows:

(a) Make an n-dimensional array of size $16^{n}$ using the secret key *K*

  rng(*K*);

  for X(0)=0:2:15,

  for X(1)=0:2:15,

  …

  for X(n−1)=0:2:15,

    $V=\mathrm{randperm}\left(2^{n}\right)-1;$

    k=0;

    for $X^{\prime }\left(0\right)=0:1,$

    for $X^{\prime }\left(1\right)=0:1,$

    …

    for $X^{\prime }\left(n-1\right)=0:1,$

      M($X^{\prime }\left(0\right),X^{\prime }\left(1\right),\;\ldots ,X^{\prime }\left(n-1\right))=V\left(k\right);$

      Mk=k+1;

    end

    …

    end

    end

  end

  …

  end

  end

(b) Repeat the array to obtain NMSM

  for X(1)=0:16:255,

  for X(2)=0:16:255,

  …

  for X(n)=0:16:255,

    M(X(0):X(0)+15,X(1):X(1)+15, …,X(n−1):X(n−1)+15)

            =M(0:15,0:15, …,0:15);

  end

  …

  end

  end

## Appendix C

  The pseudo code for fast algorithm of inner embedding is given as follows:

Input: n LSBs of secret segment $s_{j}^{L}$, basic structure for embedding

            $A=M\left(G\left(p_{X\left(0\right)i},d_{n}^{j}\right),G\left(p_{X\left(1\right)i},d_{n+1}^{j}\right),\ldots ,G\left(p_{X\left(n-1\right)i},d_{2n-1}^{j}\right)\right)$

Output: stego pixel values $\left(p_{X\left(0\right)i}^{\prime },p_{X\left(1\right)i}^{\prime },\ldots ,p_{X\left(n-1\right)i}^{\prime }\right)$

(a) Construct an n-dimensional basic structure B with all elements valued with $s_{j}^{L}$;

  $B=s_{j}^{L}\times ones\left(2,2,\ldots ,2_{\left(n\right)}\right)$;

(b) Using matrix operation to find the only matched element in both A and B;

  C=ismember(A,B);

(c) Project the element to all axes and obtain the coordinates for embedding;

  for k=0:n−1,

            $\left[v,p_{X\left(k\right)i}^{\prime }\right]=\max\underset{q\neq k}{\sum }C\left(:,:,\ldots ,{:}_{\left(q\right)},\ldots ,:\right);$

  end

  The algorithm utilizes an array of unique value $s_{j}^{L}$ to compare with the basic structure for embedding A using the function ismember(). As a result, the unique ‘1’ in the array C indicates the location of $s_{j}^{L}$ in A. By projecting the summations to each axis can obtain the index of the corresponding axis. Thus, the values of stego pixels are determined.
